# A Population-Based Study of Secondary Prostate Cancer Risk after Radiotherapy in Male Patients with Rectal Cancer: A Retrospective Cohort Study

**DOI:** 10.3390/medicina55040104

**Published:** 2019-04-14

**Authors:** Jen-Pin Chuang, Yen-Chien Lee, Jenq-Chang Lee, Chin-Li Lu, Chung-Yi Li

**Affiliations:** 1 Institute of Clinical Medicine, National Cheng Kung University College of Medicine, Tainan 70101, Taiwan; chuangjp@gmail.com; 2 Department of Surgery, Puzi Hospital, Ministry of Health and Welfare, Chiayi County 61347, Taiwan; 3 Department of Oncology, Tainan Hospital, Ministry of Health and Welfare, Tainan 70043, Taiwan; lee.gull@gmail.com; 4 Institute of Internal Medicine, National Cheng Kung University College of Medicine, Tainan 70101, Taiwan; 5 Department of Surgery, National Cheng Kung University College of Medicine, Tainan 70101, Taiwan; leejc@mail.ncku.edu.tw; 6 Institute of Food Safety, College of Agriculture and Natural Resources, National Chung Hsing University, Taichung 40249, Taiwan; chinli.lu@gmail.com; 7 Department of Public Health, College of Medicine, National Cheng Kung University, Tainan 70101, Taiwan; 8 Department of Public Health, College of Public Health, China Medical University, Taichung 40402, Taiwan

**Keywords:** rectal cancer, radiotherapy, prostate cancer

## Abstract

*Background and objective*: Risk of secondary prostate cancer after radiation therapy among patients with rectal cancer remains undetermined. Given an increased incidence of rectal cancer in younger people and improved survival for rectal cancer patients who received radiation therapy, the potential risk of secondary prostate cancer needs to be further investigated. *Materials and Methods*: Male patients (*n* = 11,367) newly diagnosed rectal cancer and who underwent abdominoperineal resection (APR) or low anterior resection (LAR) from 1 January, 1998 to 31 December, 2010 were identified from Taiwan National Health Insurance Research Database. The incidence and relative risk of secondary prostate cancer in study patients with (*n* = 1586) and without (*n* = 9781) radiotherapy within one year after rectal cancer diagnosis were compared using a competing-risks analysis. *Results*: Rectal cancer patients with radiotherapy were at a significantly decreased risk of developing prostate cancer, with a hazard ratio (HR) of 0.41 (95% confidence interval = 0.20–0.83) after adjustment for age. Analysis of the risk estimated for various follow-up lengths suggested that a decreasing HR was seen through the period followed-up and that there was a trend of decreasing prostate cancer risk with time after radiotherapy. *Conclusions*: Radiotherapy was significantly associated with decreased risk of secondary prostate cancer among rectal cancer patients, by a magnitude of 59%.

## 1. Introduction

Colorectal cancer is the third leading cancer commonly diagnosed in males, and the second in females [1] worldwide. Rectal cancer accounted for 30% of these cases [2]. In Taiwan, colorectal cancer ranked 3rd in the death and the incidence ranked first in cancer categories in 2016. With a longer survival rate and an increasing percentage of a younger population among incident cases [3], the long-term effects of colorectal cancer treatments on risks of complications are noteworthy. It has been well-established that the subsequent risk of neoplasm remains elevated for more than 20 years of follow-up in patients with various sites of cancer, including colorectal cancer [4]. Such increased risks of subsequent neoplasm are partly attributable from radiotherapy [4]. It was estimated that second cancers, including those likely to be caused by chemo- or radiotherapy, accounted for approximately 15% of all cancers registered [5].

Radiotherapy has been the standard adjuvant treatment for T3/N (+) rectal cancer [6]. Compared to those who had surgery alone, rectal cancer patients who had preoperative or postoperative radiotherapy experienced a lower one-year risk of local recurrence by 46% and 37%, respectively. However, the overall survival was only marginally better (62% vs. 63%) for adjuvant radiotherapy [6]. Recently, with the addition of chemotherapy, chemoradiotherapy has become a standard treatment for rectal cancer, which enhances pathological response and improves local control [7]. Despite the benefit in disease-free and overall survival associated with radiotherapy [8], our previous meta-analysis showed there were contrary results of protection or increased risk of prostate cancer after radiotherapy of rectal cancer patients [9] from two articles [10,11] before 3 March, 2015. Most of the studies are from the SEER population-based data [9]. Although the results showed that the potential prostate cancer risk from radiotherapy was small [10], a long-term risk in rectal cancer survivors should not be overlooked. In last year, two articles related to this topic were published, all with the same results of protection of prostate cancer incidence [12,13]. In this study, we recruited a nationally representative cohort of rectal cancer patients to investigate whether rectal cancer patients who received radiotherapy are at a greater risk of prostate cancer after a maximum of 13 years of follow-up.

## 2. Patients and Methods

### 2.1. Data Sources

The data analyzed in this study were retrospectively retrieved from the claims of the National Health Insurance Research Database (NHIRD) provided by the National Health Insurance Administration (NHIA), Ministry of Health and Welfare of Taiwan. The NHIRD provided all inpatient and ambulatory medical claims for around 99% of Taiwanese people [14], the NHIRD covered all inpatient and outpatient claims and medical orders, as well as the information on health care providers, including medical institutions and health care workers. The identification numbers of all health care providers are encrypted to ensure privacy. To ensure the accuracy of claim files, the NHIA performs expert reviews quarterly on a random sample for every 50 to 100 ambulatory and inpatient claims [15]. In Taiwan, the NHIA issues major illness/injury certificates to all patients who suffer from malignant neoplasm and these patients are exempt from copayment to the National Health Insurance (NHI) if they are admitted for the illness associated with the related malignancy. A major illness/injury certificate can be issued only when the cancer diagnosis is pathologically confirmed. To ensure the accuracy of the diagnosis of malignant neoplasm, we enrolled only those patients with rectal cancer by using major illness/injury certificates for the particular admissions or ambulatory cares. The data analyzed in this study included no personal identifiers, and thus the informed consent and review by the Institutional Review Board were waived.

### 2.2. Study Cohort

We included freshly diagnosed rectal cancer without previous radiation therapy or prostate cancer history. Due to the lack of stage information, an operation of abdominal perineal resection or anterior resection patients who had received radiation therapy within one year of rectal cancer diagnosis were included. The outcome was prostate cancer incidence with at least 1 year of rectal cancer diagnosis. Death was treated as a competing risk. The details of inclusion methods are as follows.

**Figure 1:**
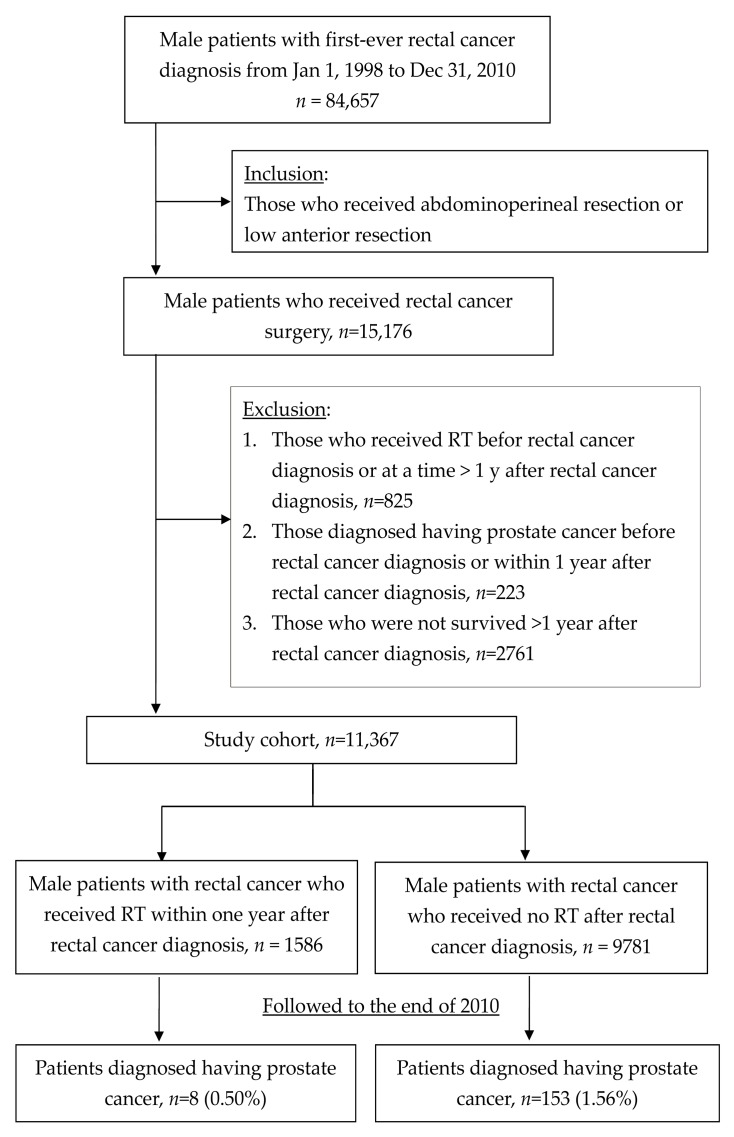
Flow chart of selection and follow-up of study participants.

Between 1 January, 1998 and 31 December, 2010, a total of 84,657 male patients newly diagnosed with rectal cancer (as per the International Classification of Disease, 9th Revision, Clinical Modification, ICD-9-CM codes: 154.0, 154.1, 154.8 or 230.4) were selected. Among them, 15,176 received definite surgery (NHIRD medical order codes: 74205B, 74206B, 74211B, 74213B, 74214B, 74216B, 74217B, 74220B, 74220A or 74222B). Radiotherapy within one year of the rectal cancer diagnosis date (MHIRD medical order codes: 36004B, 36005B, 36006B, 36009B, 36010B, 36011B, 36012B, 36012BA, 36012BB, 36012BC and 36013B) were selected. Those who received radiotherapy (RT) before rectal cancer diagnosis or at a time >1 year after the rectal cancer diagnosis, *n* = 825 were excluded. Prostate cancer patients (ICD-9-CM code: 185, 233.4, or 236.5) with diagnosis before rectal cancer or within one year of the rectal cancer diagnosis date were excluded as well (*n* = 223). The patients who did not survive >1 year after rectal cancer diagnosis were also excluded (*n* = 2761). The remaining 11,367 patients were further separated into two study groups according to whether they had undergone radiotherapy within 1 year after rectal cancer diagnosis. All study subjects were followed up from the date of rectal cancer diagnosis to the occurrence of prostate cancer, withdrawal from the NHI insurance, or the last day of 2010 (whichever came first). The prostate cancer was determined by the discharge diagnostic codes of malignant neoplasm of the prostate (ICD-9-CM code: 185), carcinoma in situ of the prostate (ICD-9-CM code: 233.4) and neoplasm of uncertain behavior of prostate (ICD-9-CM code: 236.5). This is depicted in the flow chart illustrated in Figure 1. Until the end of the follow-ups at 2010, 8 patients at the study group developed prostate cancer (0.5%) and 153 patients from the control group developed prostate cancer (1.56%). Due to the higher mortality rate compared with prostate cancer incidence, a competing risk model was used and a competing risk regression was used to adjust for other factors.

### 2.3. Statistical Analysis

Competing-risks survival analysis to compare the simultaneous risks of prostate cancer or any death was used. The survival time was calculated from the date of rectal cancer diagnosis to the occurrence of prostate cancer, withdrawal from the NHI insurance, or the last day of 2010 (whichever came first). The Fine and Gray model was used [16], which is a semi-proportional subdistribution hazard model that provides the cumulative incidence (or subdistribution) of each event of interest (new diagnosis of prostate cancer or any death), while simultaneously considering the competing risk of the other outcome. Thus, people who died before prostate cancer occurred were not censored in a way that might bias the estimates, as was possible in a Cox cause-specific analysis. For the multivariable analysis, we included age and radiation therapy. We created cumulative incidence function curves to illustrate the probability of prostate cancer or any death over the study period. We plotted the overall cumulative incidence of the 2 events against months from 1 year following the new diagnosis of rectal cancer and compared the radiation therapy effects using Gray’s test. We performed statistical analyses with STATA (StataCorp LP, College Station, TX, USA), and a two-sided *p* < 0.05 was considered statistically significant.

## 3. Results

The cohort consisted of 11,367 rectal cancer patients with 1586 patients (13.95%) receiving radiation therapy. The patient characteristics are depicted in Table 1. The median follow-up was 24 months (interquartile range of 9.7–48.3) in the RT group and 34.6 months (interquartile range 14.4–68.6) in those without RT. Those receiving RT appeared younger with a median age of 60 years (range of 15–93) than those without RT, with an age of 66 (range of 14–106). Those receiving RT therapy were more likely to receive abdominoperineal resection (30.3%), compared those without RT (19.9%). At the end of the follow-up, eight patients (0.5%) in the radiotherapy group and 153 patients (1.56%) in the control group developed prostate cancer. As expected, as age increased, the prostate cancer risk increased (Table 2). After adjusting for age, radiotherapy was associated with a significantly decreased risk of prostate cancer (hazard ratio, HR = 0.41; 95% confidence interval, CI = 0.20–0.83, *p* <0.013). This was confirmed in a competing risk model using death as the competing risk factor.

**Figure 2:**
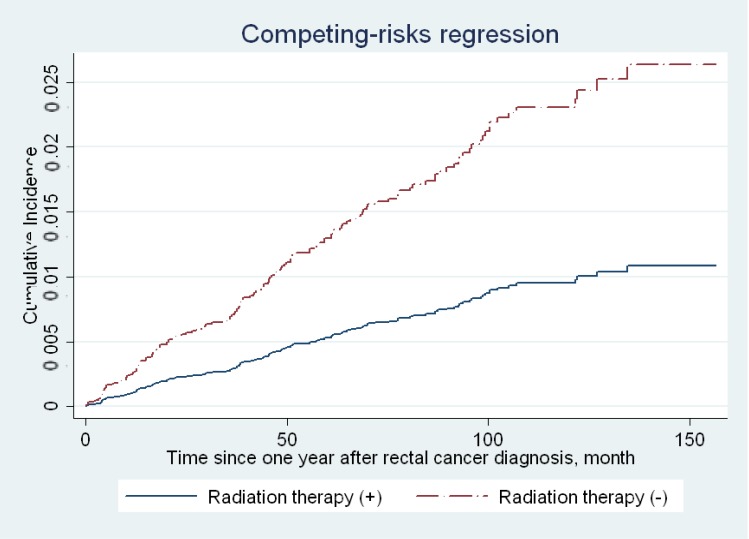
Cumulative incidence function curves for receiving radiation therapy status.

Further analysis of the risk estimated for various lengths of follow-up suggested a decreasing trend of HRs were noted with time. Radiation therapy seemed to protect the occurrence of prostate cancer as time went by. However, none reached statistical significance (Table 3). Also, as time went by, there was a trend of decreasing prostate cancer incidence (Figure 2). Our study patients were relatively young and with a lower risk of prostate cancer at the initiation of follow-up. The risk of prostate cancer increased as the ages of study patients increased (Table 2).

## 4. Discussion

This was the first large Asian general population-based study, which is in concordance with the results of a previous SEER data-based study [11] or previous Netherlands population-based cancer registry [13]. Competing risk regression model adjusted for death was used in our study to show a decreasing risk of prostate cancer after radiation therapy, compared with no radiation therapy in the rectal cancer patients group. Due to a higher incidence of death compared with prostate cancer incidence, we believed that this method was more suitable. With a relatively large sample size and long follow-up period, this present study showed that radiotherapy for rectal cancer was significantly associated with decreasing risk of secondary prostate cancer by 59%. Our study patients were relatively young and with a lower risk of prostate cancer at the initiation of follow-up. The risk of prostate cancer increased as the ages of study patients increased (Table 2). This could have partly explained the small difference in prostate cancer risk between the RT and control group, and the protective effect tended to be more obvious as the follow-up period increased. However, the information about the TNM staging, tumor grade, RT dosages and fractionation is missing in our database.

Traditionally, it is assumed that a secondary neoplasm occurs approximately five years after radiation therapy [17]. The latent period for the development of leukemia is reported to be in the range of 5–10 years, and about 10–60 years for a solid tumor [17]. This was calculated from the A-bomb explosion in 1945 [17]. Although the potential carcinogenetic side effects of radiotherapy have mainly come from evidence on survivors of atomic bomb blasts in Japan [18], there has been no definite agreement about the time period most relevant to the induction of a second malignancy. A recent population study showed that there was a significant increased relative risk of developing myeloid leukemia at 1–5 years (relative risk, RR = 2.99, 95% CI = 1.13–9.33) in breast cancer patients who received radiation therapy [19]. Our data (Table 3, Figure 2) showed that as time went by, there was a trend of decreasing prostate cancer incidence. Rombouts et al. showed that RT reduced the cumulative incidence of second pelvic tumors compared with patients who did not receive RT, which was also confirmed by multivariable analysis (*p* = 0.04) [13]. Lower secondary cancer incidence within five years of follow-up was observed compared with the RT versus non-RT group. However, after excluding prostate cancer incidence, there was no longer a significant relationship between the occurrence of second pelvic tumors and previous RT, which might suggest the protective effects of secondary prostate cancer on the RT group [13]. Warschkow et al. [12] had shown a decreasing secondary cancer risk after radiation therapy of rectal cancer versus no the RT group, even within a five-year follow-up. The decreasing risk of a secondary malignancy was largely contributed by the decreasing risk of secondary prostate cancer (HR = 0.42, 95% CI = 0.36–0.48, *p* < 0.001) [12]. While acknowledging secondary cancer incidence after five years, a secondary malignancy within five years of radiation therapy should also be emphasized, especially among rectal cancer patients with mostly older patients and less life expectancy.

Our recent meta-analysis conducted until 3 March, 2015 [9] reported that only two studies [10,11] investigated the potential adverse effect of radiotherapy among rectal cancer patients, with ambiguous results. Kendal [11] reported that there was a decreased risk of prostate cancer among rectal cancer patients receiving radiation therapy compared with no radiation therapy, while Birgisson et al. [10] reported that there was a neutral effect from two adjuvant studies, the Uppsala Trail and the Swedish Rectal Cancer Trial. The Uppsala Trial included 471 rectal cancer patients and noted an insignificantly elevated risk of prostate cancer (RR = 2.34, 95% CI = 0.29–20.95). The Swedish Rectal Cancer Trial showed an insignificantly elevated risk of prostate cancer among 1147 rectal cancer patients (RR = 1.75, 95% CI = 0.6–5.11). Kendal WS et al. [11] had excluded patients with less than five years of follow-up, and those who developed second cancer within the first five years followed-up. There were 26.98% of patients receiving radiation therapy in the Kendal study [11]. But only 9.37% of patients received radiation therapy in our study group. This might due to different inclusion criteria. In our country, long courses of radiation therapy are generally used.

One possible mechanism of decreasing prostate cancer risk might be the castration effect of radiation. One study [20] showed that serum testosterone levels only changed marginally in patients who received external beam radiation for prostate cancer. However, data from all published prospective studies on the circulating level of total and free testosterone did not support the hypothesis that high levels of circulating androgens are associated with an increased risk of prostate cancer [21,22]. The other possible reason is that radiation therapy might cure occult prostate cancer. Radiation therapy has long been known to cause secondary malignancy, largely contributed through the Childhood Cancer Survivor Study (CCSS) with the emphasis of being the strongest independent risk factor on multivariate analysis [23]. One possible disparity of the protection results on prostate cancer might be due age. Age might play a significant role in carcinogenesis after irradiation. Patients of younger age might be more susceptible to radiation [24]. As time went by, the risk of prostate cancer incidence increased while the radiation therapy of rectal cancer decreased it.

Rectal cancer might be a marker of other cancers and vice versa. It has been reported that there was 10.7% risk of prostate cancer in colorectal cancer patients compared with 3.8% in the control [9]. Common etiologies of rectal cancer and prostate cancer were red meat, high-fat dairy, overweight, age and smoking (little effects on prostate cancer). Lead-time bias, which arises from patients being monitored more closely than the general population, resulting in more secondary cancer diagnosis. All the above factors might not influence the treatment decision of radiation therapy. Thus they might not affect the outcomes.

Our study employed a large sample number of Asian patients with rectal cancer, leading to a precise estimate of the relative risk of secondary prostate cancer. Also, we didn’t exclude the secondary prostate cancer that occurred in the first five years after radiotherapy. Our study may provide more complete information on the risk of secondary prostate cancer after radiotherapy. Such information is considered more relevant and informative to clinicians and patients in daily clinical settings. Also, some rectal cancer patients were censored due to death during the follow-up period. These deceased patients could have developed prostate cancer if they did not encounter mortality. To account for the possible biased estimate of relative risk, we alternatively performed a competing risk analysis, using the Fine and Gray method [16]. Here, there were also some limitations. First, stage information was unavailable in our database. Our study relied upon claim data and diagnostic codes to ascertain cancer patients, which was subject to disease misclassification. The potential bias arising from such potential disease misclassification would be minimal, mainly because the cancer diagnosis that appeared in Taiwan’s NHI claims should be accompanied by a major illness/injury certificate that can be issued only when the cancer diagnosis is pathologically confirmed. Also, we were unable to completely adjust for some known risk factors for prostate cancer, such as but not limited to body mass index [25]. The potential for residual confounding may not be completely excluded from this study.

## 5. Conclusions

Radiotherapy was associated with significantly decreasing risk of prostate cancer incidence among rectal cancer patients who received radiotherapy.

## Figures and Tables

**Table 1:** Characteristics of male rectal cancer patients with or without radiotherapy (RT) within one year after rectal cancer diagnosis.

	With RT (*n* = 1586)		Without RT (*n* = 9781)		*p*-Value †
	*n*	%	*n*	%	
Median age (years), range	60, (15–93)		66, (14–106)		<0.001
Surgery methods					
APR	481	30.3	1950	19.9	<0.001
LAR	1105	69.37	7831	80.1	
Follow up time (months)					
Median with inter-quartile range	24.0 (9.7, 48.3)		34.6 (14.4, 68.6)		<0.001

† Based on Wilcoxon rank sum test or Pearson’s χ^2^ test; APR, abdominoperineal resection; LAR, low anterior resection; RT, radiotherapy.

**Table 2:** Incidence rate and hazard ratio of prostate cancer in relation to radiation and age.

Variables	*n*	Person-Years (×100)	No. of Event	Incidence Rate †		Adjusted HR ‡		
				Estimate	95% CI	Estimate	95% CI	*p*-Value
Radiation								
No	9781	359.52	153	0.43	0.36–0.50	1.00		
Yes	1586	45.23	8	0.18	0.08–0.34	0.41	0.20–0.83	<0.013
Age (yrs)								
<50	1599	62.74	6	0.10	0.04–0.20	1.00		
50– < 60	2404	86.76	21	0.24	0.15–0.36	2.48	1.00–6.15	0.05
60– < 70	3213	123.46	48	0.39	0.29–0.51	3.73	1.60–8.70	<0.002
≥70	4151	131.79	86	0.65	0.53–0.80	5.21	2.28–11.90	<0.001
Total	11,367	404.75	161					

† No. of cases per 100 person-years; HR = hazard ratio; ‡ Estimated from the competing risk model that simultaneously included radiation and age in the model.

**Table 3:** Incidence rate and HRs of prostate cancer in relation to radiation according to length of follow-up.

Variables	*n*	Person-Years (×100)	No. of Event	Incidence Rate †		Adjusted HR ‡		
				Estimate	95% CI	Estimate	95% CI	*p*-Value
≤2 years								
Radiation								
No	2105	10.65	32	1.02	0.71–1.42	1.00		
Yes	473	2.83	4	0.60	0.19–1.46	0.94	0.33–2.71	0.91
≤3 years								
Radiation								
No	3746	26.31	57	0.68	0.52–0.88	1.00		
Yes	802	6.23	4	0.23	0.08–0.58	0.53	0.19–1.48	0.23
≤4 years								
Radiation								
No	5043	42.24	79	0.53	0.42–0.66	1.00		
Yes	1025	8.75	4	0.14	0.04–0.34	0.39	0.14–1.06	0.07
≤5 years								
Radiation								
No	6020	55.64	103	0.47	0.38–0.56	1.00		
Yes	1197	11.47	6	0.15	0.06–0.30	0.45	0.20–1.03	0.06
>5 years								
Radiation								
No	3761	46.04	50	0.18	0.13–0.23	1.00		
Yes	389	5.67	2	0.07	0.01–0.23	0.32	0.05–2.28	0.26

HR = hazard ratio; † No. of cases per 100 person-years; ‡ Estimated from the competing risk regression that simultaneously included radiation and age (as category) in the model.
